# Root canals shaped by nickel-titanium instrumentation with automated computerized numerical control systems

**DOI:** 10.1186/s12903-021-01841-4

**Published:** 2021-09-28

**Authors:** Liming Wang, Wenxiang Li, Yeon-Jee Yoo, Shin Hye Chung, Soram Oh, Hiran Perinpanayagam, Kee-Yeon Kum, Yu Gu

**Affiliations:** 1 School of Mechanical Engineering, Shandong University, Jinan, 250061 People’s Republic of China; 2 Key Laboratory of High Efficiency and Clean Mechanical Manufacture, Ministry of Education, Beijing, People’s Republic of China; 3 Department of Conservative Dentistry, Dental Research Institute, National Dental Care Center for Persons with Special Needs , Seoul National University Dental Hospital, Seoul National University School of Dentistry, Seoul, Republic of Korea; 4 Department of Dental Biomaterials Science, School of Dentistry and Dental Research Institute, Seoul National University, Seoul, 03080 Republic of Korea; 5 Department of Conservative Dentistry, Kyung Hee University Dental Hospital, Kyung Hee University School of Dentistry, 23 Kyungheedae-ro, dongdaemun-gu, Seoul, 02447 Republic of Korea; 6 Schulich School of Medicine & Dentistry, University of Western Ontario, London, ON Canada; 7 Department of Endodontics, School and Hospital of Stomatology, Cheeloo College of Medicine, Shandong University, Shandong Key Laboratory of Oral Tissue Regeneration & Shandong Engineering Laboratory for Dental Materials and Oral Tissue Regeneration, No. 44-1 Wenhua Road West, Jinan, 250012 Shandong People’s Republic of China

**Keywords:** Automation computerized numerical control machine, Insertion angle, Processing movement, Root canal preparation, Simulated resin root canal

## Abstract

**Background:**

To investigate the efficacy of a nickel-titanium (NiTi) file with an automated computerized numerical control (CNC) system for root canal shaping.

**Methods:**

The movement of the automated device and the insertion angle were investigated. In Experiment 1, simulated resin root canals were randomly divided into four groups (n = 20): manual downward movement using a handpiece (Group 1), vertical downward movement by CNC (Group 2), reciprocating up and down movement by CNC (Group 3), and spiral up and down movement by CNC (Group 4). In Experiment 2, five different insertion angles of the NiTi file were evaluated (n = 20). Four parameters were used to evaluate the shaping ability: change in the working length, central axis offset, curvature variation, and preparation time. Groups were compared using one-way analysis of variance (ANOVA) with significance was set at *P* < 0.05.

**Results:**

The change in central axis position in the curved part of the root canal was found to be smaller in Group 4 than in other groups (*P* < 0.05). The curvature changes and preparation time of Groups 1 and 4 were significantly reduced compared with Groups 2 and 3 (*P* < 0.05). The variation in working length and curvature in the 5° insertion angle group was significantly smaller than in the other groups (*P* < 0.05).

**Conclusions:**

A spiral up and down movement, controlled by the CNC machine, and 5° insertion angle, maintained original root canal shape more precisely than other methods.

## Background

Adequate root canal preparation is essential for endodontic treatment of pulp and periapical disease [1]. The objectives of canal preparation are the removal of infection and the creation of a smooth and continuous, funnel-shaped taper for obturation, while maintaining the shape of the root canal [2, 3]. Canal preparation is dependent on clinical expertise that is gained from years of training and practice. Preparation effectiveness and efficiency can be enhanced by automation. Canal preparation with a nickel-titanium (NiTi) rotary instrument is faster, creates less canal deviation, and reduces loss of working length and apical extrusion of debris [4, 5]. In this technique, a NiTi file with cutting edges is driven by a motorized handpiece to remove infected dentin and create a tapered preparation. The modes of movement of engine-driven NiTi instruments can considerably influence the shaping ability of the instrument. Roane et al. proposed that the use of a clockwise and counterclockwise alternating motion would improve efficiency [6]. Yared et al. proposed a reciprocating movement of the NiTi instrument at 144° clockwise and then 72° counterclockwise [7]. Recent studies have confirmed that NiTi instruments have better root canal shaping ability when they are used in a reciprocating motion rather than in continuous rotation [8, 9]. Franco et al. found that in curved canals there was more unnecessary distortion of the lateral walls when instruments were used in continuous rotation. They concluded that reciprocation was superior to continuous rotation in the preparation of simulated root canals [10]. Similarly, Hwang et al. found through micro-computed tomography that the greatest deviation of canal centers and root tips in canals occurred with continuous rotation [11].

The enhanced shaping ability of reciprocating motion is accompanied by an increase in the fatigue resistance of NiTi instruments [8, 12]. Gavini et al. showed that the fatigue resistance of Reciproc R25 instruments was enhanced in reciprocating movement compared to continuous rotation [13]. Similarly, Gambarini et al. found that the resistance to flexural fatigue of TF NiTi instruments was significantly higher in reciprocating movement than in continuous rotation [14]. Also, Zhang et al. and Kim et al. showed that the fatigue resistance and anti-torque capability of instruments were enhanced by reciprocation [12, 15].

Recent advances in automation have resulted in the emergence of computer numerical control (CNC) in medicine. While CNC has already shown potential in training and instructing dentists’ clinical operations, the application of engine-driven root canal preparation is still in its infancy. Some researchers [16, 17] investigated the influence of downward movement of NiTi files using simplified automation systems with up-down motion. To investigate the influence of geometrical parameters on the cutting force and torque, Peters [18] developed a CNC system with multi-sensors to simulate the operations of root canal preparation. Ha [19] investigated the influence of pecking movement on screw-in force and torque by programming a CNC system. These researchers had investigated canal preparation with simple movements, such as up-down movements. However, more flexible operations with various movements are required for canal preparation due to the complex structure of root canals. Accordingly, we used a 3-axis CNC machine, which can be programmed to simulate various movements in root canal preparation, such as pecking, spiral and customized motions.

The aim of this study was to use a CNC machine to determine optimal methods for root preparation, thereby developing mechanical automation for root canal therapy. The study determined CNC efficacy and ease of use, and demonstrated the feasibility of automation for clinical procedures.

## Methods

### Materials and equipment

**Fig. 1:**
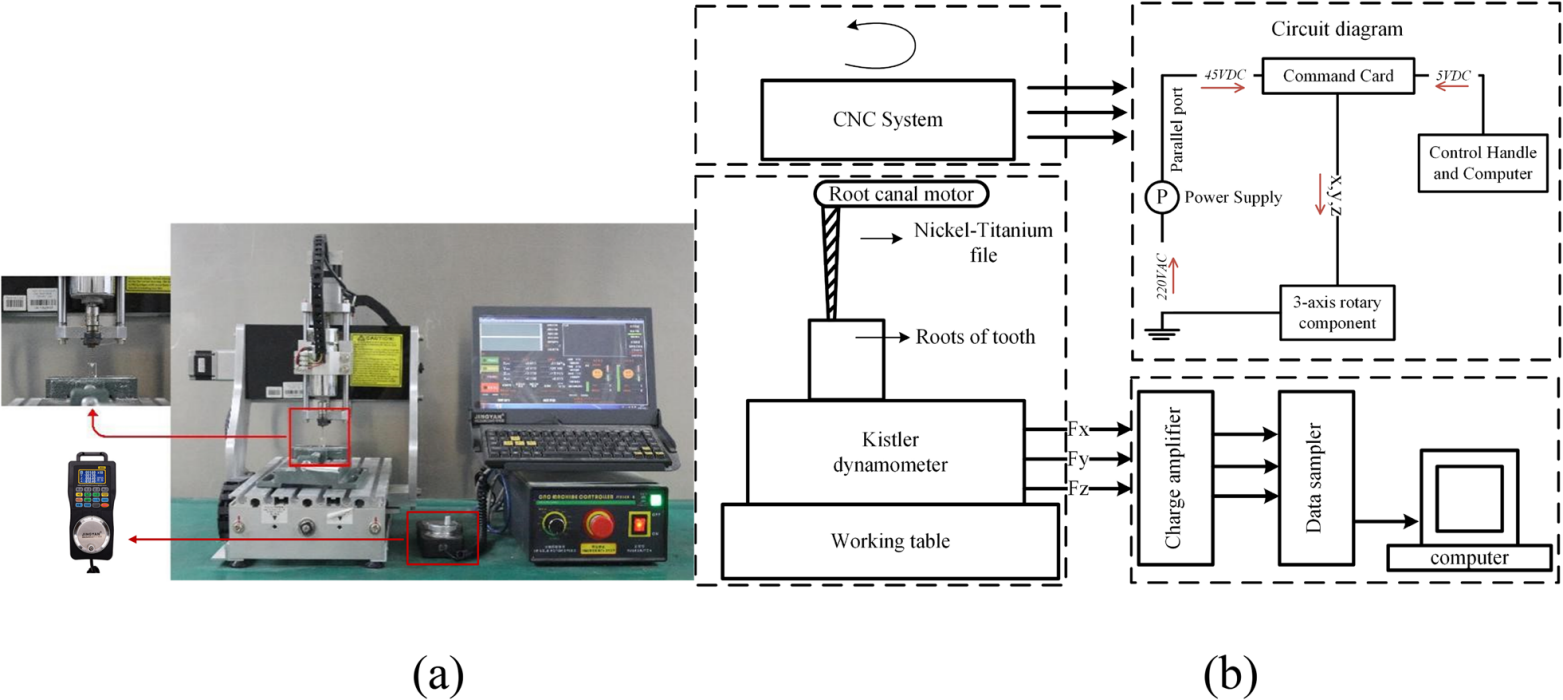
Equipment of CNC machine (**a**) and its circuit diagram (**b**)

Simulated resin curved root canals (Endo Training-Bloc, 0.02 taper; Dentsply Maillefer) were obtained (n = 180). Each canal was checked for length (18 ± 2 mm), curvature (34 ± 5°) and apical size (0.15 ± 0.02 mm). The CNC machine (CNC3020-800 W, JINGYAN, China) was applied to control the movement of NiTi files. Specifically, the maximum spindle speed of CNC was 24,000 rpm, the machining precision ranged from 0.02 to 0.05 mm, the working power and voltage were 800 W and 220 V, respectively (Fig. 1a). The circuit diagram of CNC machine was provided in this work which could achieve 3-axis motion with automatic programming [20] (Fig. 1b). A micromotor handpiece was used to drive the NiTi file for the control group in Experiment 1. The NiTi instruments were M3-Pro files (United Dental Group, Changzhou, China. n = 36 sets). Each set of M3-Pro files included MP0 (size 17, 0.12 taper), M path file (size 20, 0.02 taper), MP-1 (size 20, 0.04 taper), MP-2 (size 25, 0.04 taper) and MP-3 (size 25, 0.06 taper) for canal preparation (Table 1). Each new file was used for the preparation of only 5 simulated resin canals. The stainless-steel instruments used for measuring working length and clearing canals were #10 and #15K-files (Dentsply Sirona, York, PA, USA).

**Table 1:** Specification of NiTi files used in this study

Type	MP0	M path file	MP-1	MP-2	MP-3
Taper	.12	.02	.04	.04	.06
Size	17	20	20	25	25
Spindle speed (rpm)	300	350	350	350	350
Torque (Nmm)	3.0	1.5	1.5	1.5	2.0

### Root canal cleaning and scans

Each canal was flushed with a syringe of distilled water, dried, and then injected with black ink. The black-stained canals were scanned individually with a scanner (1200 dpi; HP G4050; China) in a standardized orientation, and stored as JPEG images. Each canal was then rinsed with distilled water to restore its transparency. Following root canal preparation, each canal was flushed with a syringe of distilled water, dried, and then injected with red ink. The red-stained canals were scanned individually with a scanner in the identical standardized orientation as before.

### Experiment 1: Instrument preparation methods

**Fig. 2:**
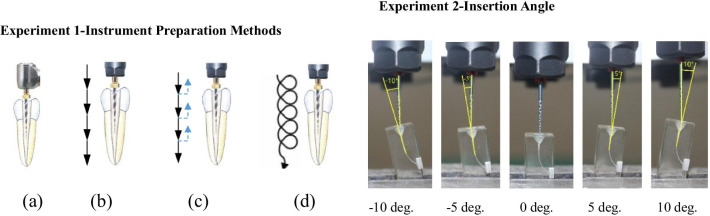
Movements for instrumentation. **a** Downward (apical) movement by handpiece; **b** Downward movement by CNC; **c** Reciprocating up (coronal) and down movement by CNC; **d** Spiral up and down movement by CNC; Angles of insertion for each group

Root canals (n = 80) were randomly divided into four groups (20/group) for preparation by each method (Fig. 2). These were downward (apical) movement using the handpiece (Group 1), downward movement by CNC (Group 2), reciprocating up (coronal) and down movements by CNC (Group 3), and spiral up and down movements by CNC (Group 4).

The instrumentation sequence was as follows: (1) #15K-file adjusted to working length using the handpiece; (2) MP0 (size 17, 0.12 taper) as an orifice shaper (300 rpm and 3.0 N cm torque); (3) Mpathy (size 20, 0.02 taper) for passageway (350 rpm and 1.5 N cm torque); (4) MP-1 (size 20, 0.04 taper; 300 rpm and 3.0 N cm torque), MP-2 (size 25, 0.04 taper; 350 rpm and 1.5 N cm torque) and MP-3 (size 25, 0.06 taper; 350 rpm and 2.5 N cm torque) to working length.

Group 1 (downward movement by handpiece): A rotating NiTi file was passed into the canal to the predetermined length, with pressure applied to the outer canal wall.

Group 2 (downward movement by CNC): A rotating NiTi file was passed into the canal at 1.5 mm/s to the predetermined length.

Group 3 (reciprocating up and down movement by CNC): A reciprocating NiTi file was passed into the canal in a pecking motion of 2 mm/s downward and 0.5 mm/sec up, until it reached the predetermined length.

Group 4 (spiral up and down movement by CNC): A rotating NiTi file was passed in a helical motion into the canal at a speed of 1.5 mm/s, while revolving around the canal in a circle with a radius of 0.1 mm, at 0.2 mm/s, until it reached the predetermined length.

### Experiment 2: Insertion angle

The spiral movement was chosen as the preparation method in experiment 2 due to its excellent performance in experiment 1. The same operation parameters in Experiment 1 Group 4 were adopted for Experiment 2, except for the angle of insertion. In the clinical practice of root canal preparation, instruments are usually inserted into the canal in an angled orientation. This angle of inclination between the direction of the instrument and the axis of the canal was described as the angle of insertion. The angles of insertion chosen were − 10°, − 5°, 0°, + 5° and + 10°, which were either along (−) or against (+) the canal curvature. An angle of 0° served as the control (Fig. 2). The canals (n = 100) were randomly divided into each of the 5 groups (20/group) for instrumentation. To eliminate errors caused by complicated pathways of movement, the CNC downward movement of NiTi files was chosen for experiment 2.

### Evaluation of canal preparation

To assess the effectiveness of the canal preparation methods, 5 parameters were chosen for evaluation. These were changes in working length, central axis offset, changes in curvature, and the length of time for canal preparation.

#### Working length

Before canal preparation, a #10K-file was used to dredge the curved root canal, and the file tip was positioned flush with the apical foramen, under 10× magnification (BC4K; BOCHENG; China). This working length was measured 3 times for each sample, and the average value recorded as WL1. Following canal preparation, the working length was measured by the same process and recorded as WL2. The change in working length was calculated as, ΔWL = WL1 − WL2.

#### The offset of central axis

**Fig. 3:**
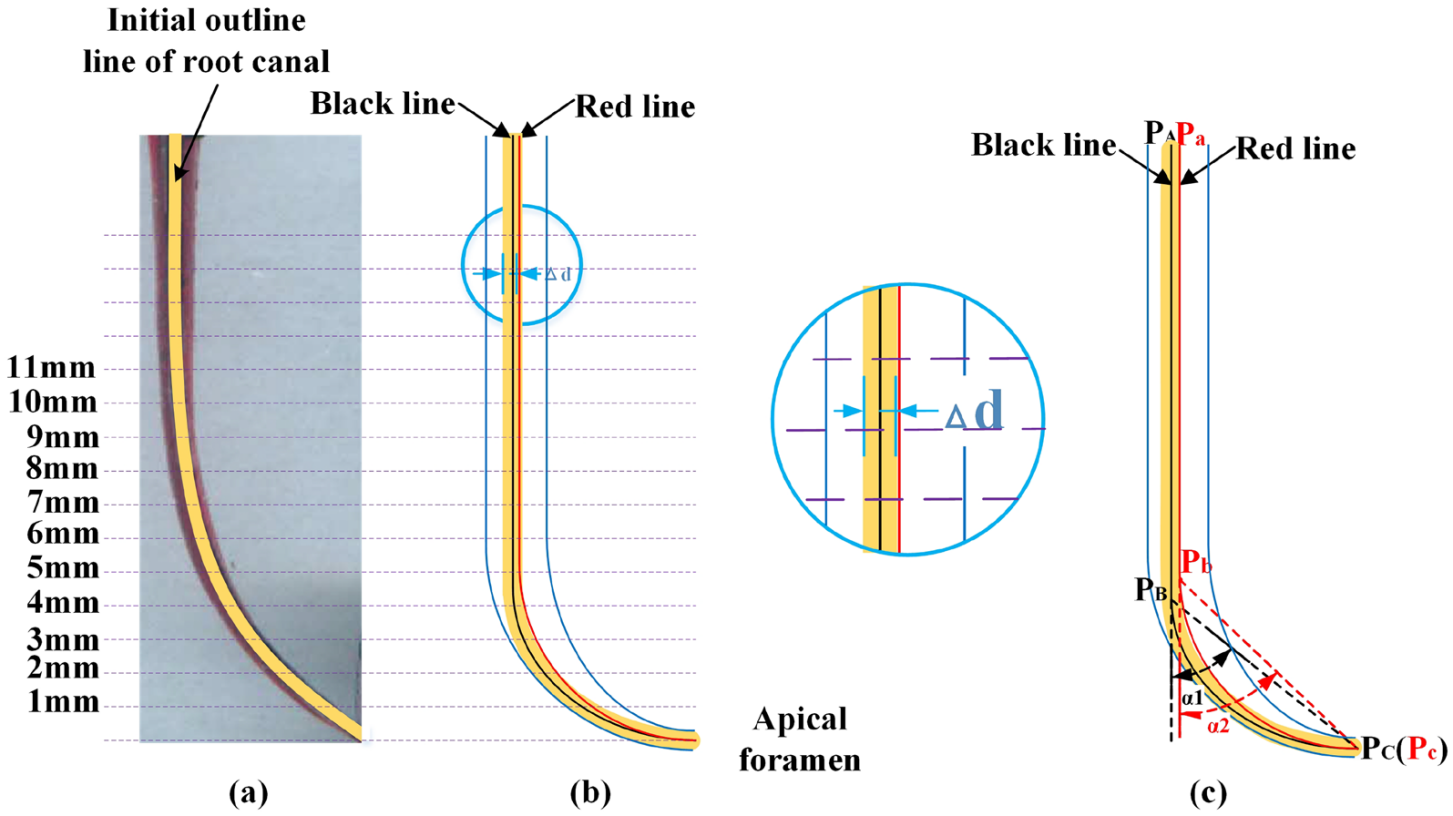
Overlapping images before and after root canal preparation and the measurement of central axis and curvature **a** the overlapping images of experiment. Yellow outline: outline of root canal before canal preparation. Red outline: outline of root canal after canal preparation; **b** the schematic diagram of central axis; **c** the schematic diagram of curvature). Black line: The central axis of the root canal before preparation. Red line: The central axis of the root canal after preparation

Canal images before and after preparation were superimposed using Adobe Photoshop CS software (Adobe system Inc, San Jose, CA, USA) to measure the offset of the root canal central axis. The transparent structure of resin materials makes it difficult for direct scanning before and after working. Thus, to represent the outline of root canal clearly, the red and black ink were selected to distinguish canals before and after preparation. A series of points at 3 mm, 7 mm, and 11 mm intervals along the canal axis were selected to evaluate the offset (Fig. 3a). The first point was 1-mm away from the apical foramen. The offset of the two central axes, denoted as $\Delta \mathrm{d}$ in Fig. 3b, was measured using ImageJ software (Media Cybernetics Inc, Rockville, MD, USA).

#### The variation of curvature

Using the Schneider method [21], the position of the apical foramen before and after preparation are marked as point P_A_ and P_a_, respectively (Fig. 3c). Following this, the points where the canal bends to a curve are marked as P_B_ and P_b_, and the apical foramen are marked as P_C_ and P_c_. The acute angle α1 and α2 formed by P_A_ P_B_ P_C_ and P_a_ P_b_ P_c_ are defined as the curvature of the root canal. Here, ImageJ software was used to measure the curvature of the simulated root canal before and after endodontic preparation. The curvature of each sample was measured 3 times and the variation in curvature along the root canal was calculated as the difference between α1 and α2.

#### Root canal preparation time

A timer was used to record root canal preparation time using each NiTi file method, from beginning to the end of the canal, excluding time for irrigation and instrument replacement.

#### Success rate

Successful cases were defined as those with a working length varying by less than 0.5 mm, a curvature varying by less than 5°, and a central axis offset less than 0.4 mm after root canal preparation. Absence of NiTi file separation, perforation, or ledge formation were also necessary for the case to be deemed successful.

### Statistical analysis

SPSS 22.0 (SPSS Inc., Chicago, IL, USA) statistical analysis software was used to process the measurement data, which were expressed as mean and standard deviation for each group. One-way analysis of variance (ANOVA) and *P*-values were used to compare experimental groups (Tables 2, 3).

**Table 2:** Variation of root canal after preparation by 4 processing movement (mean ± SD)

Groups	The variation of root canal after preparation				
	Working length (mm)	The mean of offset on central axis (mm)	Curvature (°)	preparation time (s)	Success rate (%)
Group 1	.20 ± .16	.085 ± .040	2.12 ± 1.04	83.25 ± 15.31	90
Group 2	.32 ± .11	.137 ± .050	3.75 ± 1.37	52.32 ± 3.84	80
Group 3	.26 ± .11	.104 ± .043	3.02 ± 1.48	124.51 ± 6.17	85
Group 4	.18 ± .12	.075 ± .035	2.12 ± 0.95	55.42 ± 4.64	90
*P*-value	0.021	0.010	0.028	0.039	N/A

**Table 3:** Variation of root canal after preparation at different insert angles (mean ± SD)

Groups	The variation of root canal after preparation				
	Working length (mm)	The mean of offset on central axis (mm)	Curvature(°)	preparation time (s)	Success rate (%)
Group − 10°	.41 ± .26	.247 ± .041	4.22 ± 1.06	59.41 ± 6.33	75
Group − 5°	.34 ± .17	.145 ± .042	3.86 ± 1.21	52.62 ± 7.84	85
Group 0°	.20 ± .12	.085 ± .032	2.54 ± 0.87	54.58 ± 7.15	90
Group 5°	.17 ± .15	.073 ± .025	2.08 ± 0.93	53.89 ± 6.64	90
Group 10°	.37 ± .36	.119 ± .035	3.59 ± 1.34	51.33 ± 8.60	80
*P*-value	0.025	0.018	0.031	0.030	N/A

## Results

For canals (n = 80) in Experiment 1, there was one ledge and one root perforation in Group 1, two cases of NiTi file separation and two root perforations in Group 2, one file separation and two cases of ledging in Group 3, and one ledge and one perforation in Group 4. Group 2 had the most iatrogenic events, whereas Groups 1 and 4 had the fewest.

The working length before and after root canal preparation varied for the four methods (Table 2). Clinical requirements were that working length should be between 17.5 and 18.5 mm after preparation. There were no significant differences in working length between groups (*P* < 0.05). Group 2 showed the largest variation, and Group 4 the least.

**Fig. 4:**
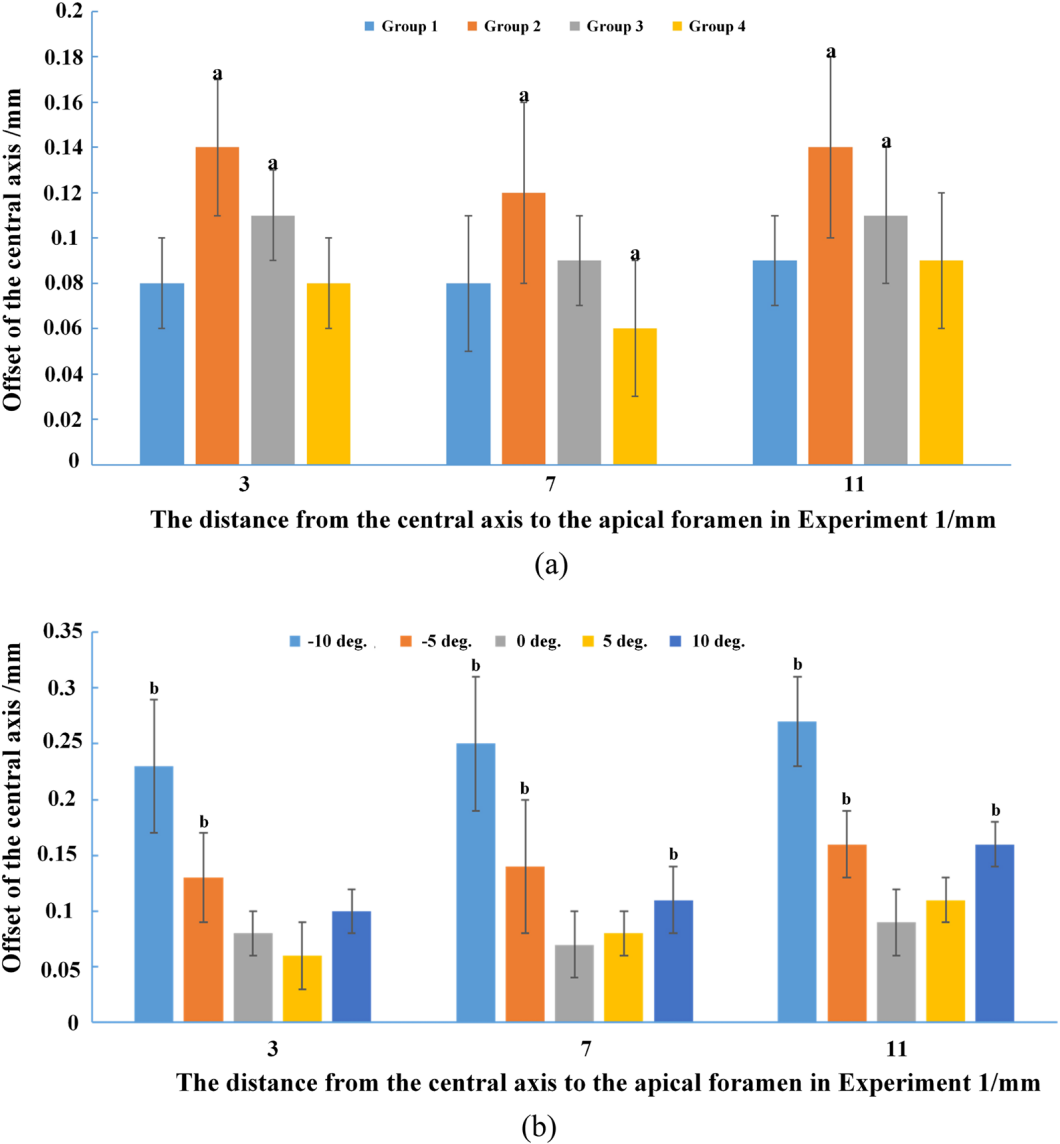
The offset of the central axis before and after root canal preparation using the four preparation methods and various insertion angles: **a** Significant difference between the four preparation methods; **b** Significant difference between the insertion angles. Horizontal axis means the distance from the apex of root canal, which was used to evaluate the offset of root canal after preparation

The images taken before and after root canal preparation were superimposed (Fig. 3a). There was offset of the central axis before and after root canal preparation in Experiment 1 (Table 2) as measured at the 3 data points (Fig. 4). The horizontal axis (Fig. 4) represented the distance from the apex of root canal, which was used to evaluate the offset of root canal after preparation. The offset of the central axis at each point in Group 1 and Group 4 was significantly less than that in Group 2 and Group 3. The offset of the central axis of Group 4 was less than that of Group 1 at the data points 4–7 mm away from the root canal apex (*P* < 0.05).

There was variation in curvature before and after root canal preparation in Experiment 1 (Table 2). Generally, the curvature should be controlled between 34 and 36° in a clinical setting. The curvature variation after root canal preparation for Groups 1 and 4 were significantly smaller than that for Groups 2 and 3 (*P* < 0.05), which means that different factors were having significant effects. There was no significant difference in curvature variation between Group 1 and Group 4.

The root canal preparation times for Experiment 1 (Table 3) in Groups 1 and 4 were significantly shorter than in Groups 2 and 3 (*P* < 0.05). There was no significant difference in root canal preparation time between Groups 1 and 4.

For canals (n = 100) in Experiment 2, there were two cases of NiTi file separation, one ledge, and two root perforations in the − 10° group. There was one ledge and two root perforations in the − 5° group, one ledge and one root perforation in the 0° group, one ledge and one root perforation in the 5° group, and one ledge and three root perforations in the 10° group. The − 10° group had the most iatrogenic events, whereas the 5° group had the fewest. Perforation and ledging occurred more frequently in Experiment 2 than in Experiment 1.

There was variation in working length before and after root canal preparation with different insertion angles (Table 3). The variation in working length for an insertion angle of 5° was significantly smaller than all other insertion angles (*P* < 0.05).

For each insertion angle, there was offset of the central axis before and after root canal preparation (Table 2, Fig. 3). The offset of the central axis with insertion angles of 0° and 5° were significantly less than at other insertion angles (*P* < 0.05). The offset of the central axis with an insertion angle of 5° at 3–5 mm from the root canal apical foramen was less than that at other insertion angles (*P* < 0.05).

There was variation in curvature before and after root canal preparation with different insertion angles (Table 2). The curvature variations for the 5° insertion angle group were significantly smaller after root canal preparation than in the other groups (*P* < 0.05). The *P*-values (Tables 2, 3) showed that the motion and insert angle with different factors for the root canal preparation have statistical significance.

The root canal preparation time with different insertion angles were compared (Table 2). There was no significant difference in root canal preparation time among all the groups because the same preparation method had been used.

## Discussion

This study analyzed and evaluated the root canal shaping ability of automated computer-controlled instruments, with different movements and angles of insertion, using resin simulated curved root canals. Simulated root canals are often used to compare instrumentation in canal preparation because they provide standardized and reproducible canal sizes, lengths, and curvatures. Additionally, they can be easily inspected, photographed, and measured, and intuitively reflect the changes that occur during canal preparation [10, 14, 22].

Success rates for root canal preparation have been evaluated in numerous studies. Schafer et al. [21] reported a success rate of 75% when K3 instruments were used with continuous rotation in simulated canals with a 35° curvature. Merrett et al. [22] reported a success rate of 80% using FlexMaster with continuous rotation in simulated canals. Jin et al. [23] achieved a success rate of 90% using a single RaCe file with a reciprocating motion in simulated canals. Similarly, overall success rates of 70–90% were achieved by manual operation in our study. However, higher success rates were attained by the automated CNC techniques.

The efficiency of root canal instrumentation and preparation time for standardized canals is dependent on the operator, instruments and preparation technique [23, 24]. Burklein et al. reported preparation times of 160.2 s with Mtwo rotary files [24], and 242.4 s with Hyflex CM and continuous rotation in simulated canals [9]. Jin et al. [23] achieved a preparation time of 69 s by using a single ProFile in reciprocating motion. However, all these studies reported on manually operated, engine-driven handpieces. In this study we used M3-pro files with automation technology. The preparation time in the manually operated control group was 83.25 s, which is consistent with a previous study on M3-Pro files with continuous rotation in simulated root canals that reported a preparation time of 77.29 s [25]. In contrast, the preparation time for CNC-operated instruments with a reciprocating helical motion was markedly shorter, at only 52.32 s.

The shaping ability of NiTi instruments has been evaluated by several investigators [5, 25, 26]. In this study, the shaping ability of various instrumentation movements and angles of insertion were evaluated by five criteria. These were changes in working length, changes in canal curvature, positional changes of the central axis, preparation time, and overall rates of success. In Experiment 1, both the downward movement and the reciprocating up and down movement by CNC created more enlargement by cutting on the outer surface of curvatures, which increased canal curvature and working length. In contrast, the spiral up and down movement by CNC enabled a self-adjustment of the file through its inherent flexibility, which contacted and enlarged both the inner and outer surfaces of canal curvatures simultaneously. Accordingly, the changes in working length, curvature, and central axes were the smallest of all the techniques. In Experiment 2, an angle of insertion of 0° allowed the M3-Pro file to contact and cut on the outer surface of curvatures, which created some enlargement. As the angle of insertion increased negatively, the instrument was positioned increasingly towards the outer surface of curvature, which created more enlargement and greater changes in working length, curvature, and position of the central axis. As the angle of insertion was increased positively, the instrument was positioned increasingly towards the inner surface of curvature, which reduced the enlargement and changes in the canal. However, a large positive insertion angle created excessive cutting on the inner surface of canal curvature. Indeed, these insertion angles were only maintained until the instruments reached positions of maximum curvature within the canal. Thereafter, different loading stresses and bending resistance were encountered by the NiTi files as they traversed canal curvatures (Fig. 2b).

Ideally, these shaping techniques should not create any ledging, root perforations, or instrument separations during canal preparation [27–29]. Unfortunately, in Experiment 1, using the file in a downward movement with the CNC machine resulted in a high frequency of instrument separation and root perforation. In downward movement the file was inherently less flexible when encountering resistance within the canal. Similarly, files used with the CNC machine in the reciprocating up and down movement were prone to creating ledges and to separation, as there was considerable vibration during the up and down cycle. In Experiment 2, a large negative angle of insertion with the file positioned towards the outer surface of curvature was associated with an increase in root perforations and instrument separations.

Potentially, more perforations and NiTi file separations could have been caused by the constant downward speed of CNC-driven instruments. However, these potential pitfalls were avoided by a 10% adjustment in the downward speed that was engineered to accommodate instruments that encountered canal blockages or excessive vibration. These slowdowns occurred during certain movements and angles of insertion for canal preparation, which increased preparation times and reduced efficiencies.

Within the limitations of this in vitro study, the use of a NiTi file driven by a CNC machine in a spiral up and down motion with a 5° angle of insertion was found to be the most efficient method for root canal preparation while maintaining the original shape of the canal. This model utilizing computer-controlled instrumentation and resin curved root canals was highly standardized and reproducible. Furthermore, this automated technology could be developed for clinical applications in root canal therapy.

## Conclusions

This automated computer-controlled instrumentation of resin simulated curved root canals demonstrated a highly standardized and reproducible study model. Spiral up and down movements with a 5° angle of insertion were found to be the most efficient method for root canal preparation. Furthermore, these automated techniques could be developed for applications in root canal therapy when combined with AI technology, such as the AI robotic systems for autonomous root preparation.

## Data Availability

All data generated during this study are included in this published article.

## Abbreviations

NiTi: Nickel-titanium
CNC: Computerized numerical control
